# 15000 Exploring team science, professional networks, and innovation success in the THRIVE COVID-19 fellowship program

**DOI:** 10.1017/cts.2021.680

**Published:** 2021-03-30

**Authors:** Layla Fattah, Janice Gabrilove, Holly Oemke, Joseph Borrello, Turner Baker, Kevin D. Costa, David Putrino, Anthony Costa

**Affiliations:** ISMMS

## Abstract

ABSTRACT IMPACT: Implement and evaluate a fellowship program to foster a new generation of entrepreneurial and collaboratively-minded team scientists, equipped with the knowledge and skills to innovate technology-based solutions for COVID-19 to advance human health OBJECTIVES/GOALS: Mount Sinai Targeted Healthcare Innovation Fellowship (THRIVE) is a 9-month program for participants from diverse professional backgrounds to develop HealthTech innovations related to COVID-19. The program is designed to provide an experiential team science platform for fellows to take an idea from concept to commercially viable innovation. METHODS/STUDY POPULATION: Following a competitive application process, 16 THRIVE fellows comprise four teams working collaboratively in an online forum with input from experts in the field. Success of the program will be evaluated by:

assessing pre- and post- collaborative research orientation among THRIVE fellows using the ROI scale1

using social network analysis (SNA) to investigate the social networks of THRIVE fellows to capture patterns of communication and collaboration related to innovation development

exploring participant experiences of group formation, teamwork and collaboration related to innovation development using one-to-one semi-structured interview

determining team success in innovation development, measured by number of publications, funding awarded, provisional patents and viable products. RESULTS/ANTICIPATED RESULTS: Paired t-tests will determine whether collaborative orientation of THRIVE fellows changes pre- vs. post- program participation, indicating changes in attitude toward multidisciplinary team work. SNA will be used to describe structural patterns of communication that occur at individual and group levels. Network-level indices will provide insight into patterns of communication that exist in innovation development: degree centrality (number of connections per individual), betweenness centrality (number of bridges to others in a network), closeness centrality (closeness to others in a network). We will also test for associations between network characteristics and team success. DISCUSSION/SIGNIFICANCE OF FINDINGS: Understanding patterns of formal and informal relationships, interactions, and perceptions of the collaborative process among individuals in THRIVE teams will elucidate whether such a program can provide an effective forum for team science and innovation development related to COVID-19.

